# The Pathology and Physiology of Ileostomy

**DOI:** 10.3389/fnut.2022.842198

**Published:** 2022-04-22

**Authors:** Haitao Ma, Xiaolong Li, Hua Yang, Yuan Qiu, Weidong Xiao

**Affiliations:** Department of General Surgery, Xinqiao Hospital, Army Medical University, Chongqing, China

**Keywords:** mucosal barrier, microbiota, distal dysfunctional intestine, probiotics, ileostomy

## Abstract

An ileostomy is a surgery that is commonly performed to protect low pelvic anastomoses or prevent high-risk anastomotic leakages. However, various postoperative complications remain of major concern. After an ileostomy, the distal intestinal segment is left open for an extended period and is in a non-functional state. Consequently, the intestinal mucosa, smooth muscle, and microbiota undergo significant changes that are closely related to postoperative recovery and complications. A systematic description of these changes is necessary to understand the relationship among them and take more effective measures for postoperative intervention.

## Introduction

**FIGURE 1:**
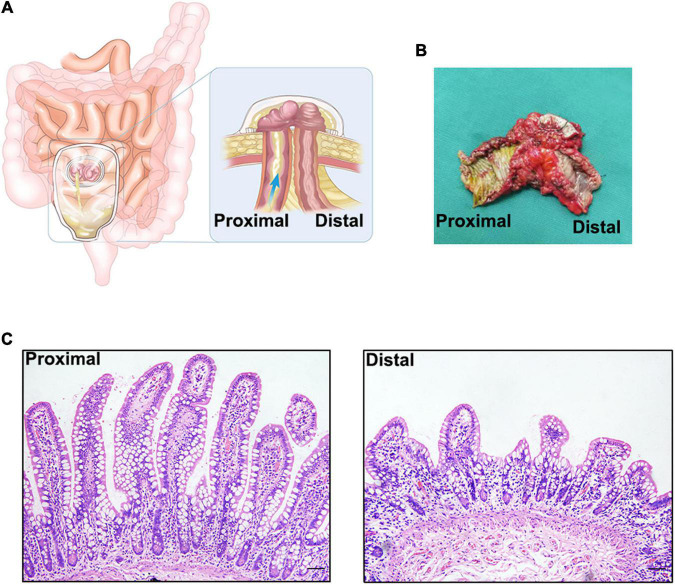
**(A)**. Schematic diagram of an ileostomy. **(B)** Ileal tissue from an ileostomy reversal, there is a significant difference between the proximal and distal intestinal tissue by visual inspection. **(C)** Hematoxylin-eosin staining of proximal and distal intestinal tissues. There is a significant atrophy in distal intestinal mucosa.

An ileostomy is when the lumen of the ileum (small bowel) is brought through the abdominal wall *via* a hole that is created during an operation (Figure 1A). It is recommended as a remedial measure in many situations, including Crohn’s disease and ulcerative colitis. It can protect and ameliorate an anastomotic leakage after a low pelvic anastomosis, or help to prevent a high-risk anastomotic leakage. With the advancement of medical technology, the anatomical position of anus-preserving surgery for low-lying rectal cancer is reducing, and there are increasing numbers of patients undergoing ileostomy. Whether in an elective setting or as an emergency, an ileostomy is considered a life-saving operative technique that maximizes the possibility of either helping save a patient’s life or improving his or her quality of life (1). However, neither clinical research nor basic research has paid enough attention to the special physiological state of ileostomy in patients.

Patients who undergo ileostomy face a series of early or late complications. Ileostomies have the highest rate of complications compared to other ostomies, with the incidence of stomal complications ranging from 21 to 70% (2). In the early stages after an ileostomy, there is a high risk of fluid and electrolyte imbalance due to a high-output enterostomy. In the late stages, fistula-related enteritis, referred to as diversion colitis, may occur. Even after the reversal of ileostomy, the patient faces problems such as slow recovery and intestinal obstruction. These problems affect the quality of life of patients and need to be urgently addressed in the clinic. Therefore, it is necessary to dissect the pathology and physiology of ileostomy.

After an ileostomy, the distal intestinal segment is left open for an extended period in a non-functional state (Figure 1B). Before the reversal of ileostomy, the distal intestinal segment loses most of its receptivity to luminal stimulation, such as mechanical stimulation, and that from intestinal microbes and their metabolites. The environment of the intestines after an ileostomy is, to some extent, similar to that seen in patients with parenteral nutrition (PN), but enteral nutrition from the proximal intestine can nourish the distal intestine through the mesentery. Thus, the two conditions are different. As a result, the state of the fistula affects the function of the intestine and even the whole body, based on the morphology, composition, and function of the intestinal mucosa, smooth muscle, and microbiota. Previous studies have referred to the mucosal and muscle layer atrophy and microbial reduction in the distal dysfunctional segment, but there is a lack of systematic description of these changes and discussion of the possible clinical relationships. Thus, this review focuses on the changes in the distal dysfunctional segment, its comparison to intestinal changes in patients with PN, and the effect of luminal stimulation on the intestine and will provide some advice for certain clinical scenarios.

## Changes in Mucosal Morphology

After an ileostomy, both the proximal and distal intestinal mucosa of the ileostomy show adaptive changes over time. Within a short period after an ileostomy, the mucosal thickness and villus height in the functional proximal segments of the intestines are significantly increased in both humans and mouse models (3–5). Mucosal hypertrophy and hyperplasia may compensate for the loss of function of the distal intestinal segment. However, after a long period after an ileostomy, these changes in the proximal intestinal mucosa may become less prominent in humans (5). Therefore, these mucosal changes may exert their effects during early adaptation, but not during late adaptation. In the distal dysfunctional intestinal segment of an ileostomy in humans, the intestinal mucosa undergoes atrophic changes in the absence of luminal stimulation (5, 6) (Figure 1C). Such atrophic changes may result from the decreased proliferation of the intestinal epithelium. RNA sequencing analysis of the distal intestinal mucosa after an ileostomy showed that the expression of genes related to proliferation, such as *Ki67*, is decreased. Wieck et al. found that although the expression of intestinal stem cell (ISC) markers, such as *LGR5*, and *ASCL2*, in the distal dysfunctional intestinal segment is increased, the number of ISCs is decreased (4). However, the preliminary results of our laboratory experiments showed that the ratio of ISCs to all epithelial cells in the distal dysfunctional intestinal segment is increased. The lack of luminal stimulation seems to minimize the impetus for ISCs to proliferate and differentiate but ISCs may have an enhanced stemness; such a state may be a physiological preparation for rapid recovery when luminal stimulation resumes. Although the cause of this special state remains unclear, a previous study confirmed that mechanical stimulation activates the Piezo1 protein to promote epithelial cell proliferation through the ERK pathway (7). Another study also demonstrated that mechanical stimulation promotes enteroendocrine cell differentiation through Piezo1 in the Drosophila model (8). Furthermore, single-stranded RNA is also sensed by Piezo1, which governs 5-HT production in enterochromaffin cells (9). Luminal stimulation including mechanical and microbial stimulation may be important signals for the proliferation and differentiation of the intestinal epithelium to maintain integrity. However, this requires further exploration.

## Changes in Transportation, Digestion, and Metabolism

In the human body, the small and large intestines have different functions. The small intestine is the key to the digestion and absorption of micro- and macro-nutrients. The large intestine is crucial for absorbing water, allowing proper defecation, and harboring intestinal microbes. Together, they form an important constituent of the body’s digestive tract. However, ileostomy destroys intestinal continuity. As a result, the function of the intestines is altered significantly.

A key function of the large intestine is the absorption of water and salts; the ileostomy abolishes the entire function of the large intestine. In compensation, the function of the proximal intestinal segment increases. Clinical research has demonstrated that daily output after ileostomy (500–700 ml) is lower than the predictive value (1,000–1,500 ml) in most cases, and potassium excretion and sodium retention are significantly increased (10, 11). This may be due to changes in circulating aldosterone levels and mineralocorticoids. These hormones are elevated after ileostomy and are decreased again after loop ileostomy reversal (12, 13). In addition, in the ileal mucosa in a model of rat colectomy, the mRNA levels of epithelial sodium channel (ENaC) proteins were significantly increased compared with unoperated controls (14). Expression levels of ENaC proteins were also elevated in non-colectomized aldosterone-infused rats (15). An aldosterone-induced increase in sodium glucose co-transporter 1 (SGLT1) activity was noted in colectomized rats (15). The loss of function of the large intestine may lead to a decrease in the sodium absorption and a temporary decrease in blood sodium levels, which in turn leads to an increase in corticosteroids. Consequently, the sodium absorption function of the proximal intestinal segment is enhanced to adapt to an ileostomy and to avoid ileostomic diarrhea. In contrast, the capacity for transportation in the distal mucosae seems to be reduced. In patients who have undergone an ileostomy, both the expression of *SGLT-1* mRNA and glucose-coupled sodium transport were significantly reduced in the distal intestinal mucosa compared to the proximal intestinal mucosa (16). After performing loop ileostomies in rats, the *NHE-3* (coding Na (+)/H(+) exchanger) gene expression was attenuated in the distal dysfunctional ileal mucosa, and the *AQP3* (a water channel) mRNA levels were decreased in the distal dysfunctional region by 30–50% (17, 18). Luminal stimulation triggers intestinal mucosal transport, which is regulated by mineralocorticoids. Therefore, although the mineralocorticoid levels at the proximal and distal segments of the ileostomy are the same, the lack of luminal stimulation of the distal intestinal cavity reduces the transportation capacity of the distal segment.

In addition to transportation capacity, digestion and metabolism also change in the absence of luminal stimulation. In a clinical trial of patients with loop ileostomy, analysis of RNA sequencing showed that genes of the distal dysfunctional intestine that are associated with digestion, nutrient transport, and absorption were significantly upregulated, particularly those for fatty acids and cholesterol, which are over-represented pathways (4). Such changes may promote adaptation after ileostomy. The absorption area for vitamin B12 is limited to the ileum, and the reabsorption of bile acids takes place in the terminal ileum; an ileostomy may cause vitamin B12 and bile acid malabsorption (19), but this is yet to be confirmed. In addition, L-cells (one of the enteroendocrine cells) secrete peptide YY (PYY), which plays an important role in decreasing gastric emptying, pancreatic secretions, and small bowel motility; the net effect being the optimization of gut absorption (20). Previous studies have indicated that PYY secretion depends on the sensing of luminal nutrients, such as short-chain fatty acids (SCFAs) and bile acids (21, 22). Oh et al. found that mucosal PYY content in the distal dysfunctional mucosa was significantly lower than that in the proximal function ileum in the short term, but the two were similar in the long term (5). The reduction of PYY in the distal dysfunctional mucosa may result from loss of luminal stimulation, but this effect may be reversed by other body factors over a long period. Without luminal stimulation, the intestinal functional spectrum of digestion, metabolism, and transportation undergoes adaptive changes, but how the changes take place still requires further exploration. Some omics methods, such as plasma metabolomics and local proteomics, may be used to systematically study the effects of an ileostomy on the intestines and body.

## Changes in the Intestinal Mucosal Barrier

The gastrointestinal tract, which is exposed to countless microbes and environmental antigens, is the largest immune organ in the body. It forms a complex and complete mucosal barrier to prevent the invasion of foreign antigens. The mucosal barrier is mainly composed of chemical barrier, mechanical barrier, immune barrier, and biological barrier (23).

**FIGURE 2:**
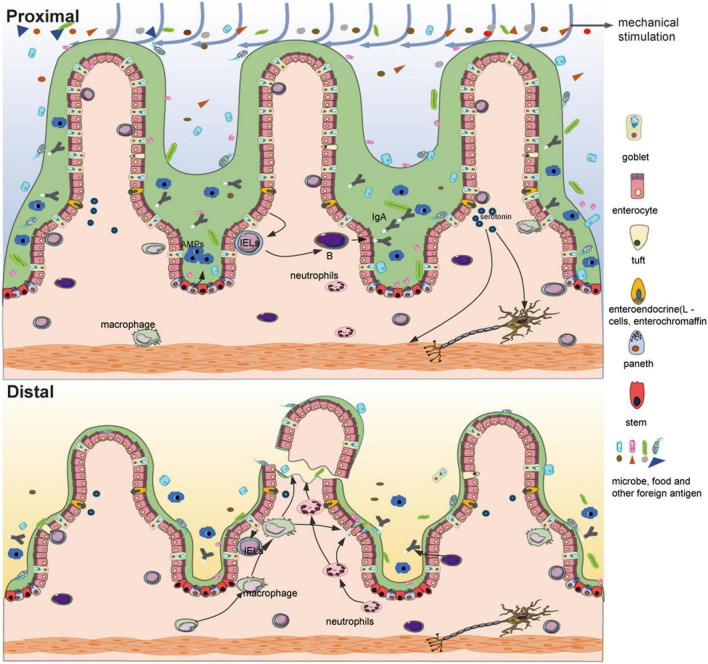
Differences between the functional proximal intestinal segment and the non-functional distal intestinal segment of an ileostomy. Compared with the proximal intestinal segment, fragile and atrophic intestinal villi, a significantly reduced number of goblet cells, thinned mucus layer, decreased Paneth cells with fewer antimicrobial peptides (AMPs), reduced mucosal bacterial load and microbial diversity, decreased intestinal interepithelial lymphocytes, aggregated innate immune cells (such as neutrophils), atrophic muscular layer and possible reduction of serotonin are shown in the distal intestinal segment.

The chemical barrier is mainly composed of chemical substances secreted by the gastrointestinal tract such as mucin, gastric acid, bile, lysozyme, various digestive enzymes, and bacteriostatic substances produced by intestinal bacteria. The intestinal mucus barrier is an important part of the chemical barrier and is mainly controlled by goblet cells. Goblet cells are a group of specialized epithelial cells that synthesize secretory mucin glycoproteins (MUC2) and bioactive molecules such as epithelial membrane-bound mucins (MUC1, MUC3, and MUC17), Fc-gamma binding protein (FCGBP), trefoil factor peptides (TFF), and resistin-like molecule beta (RELMβ) (24). These active substances, along with water and inorganic salts, form a barrier on the surface of the intestinal mucosa, which plays an important role in preventing the invasion of foreign pathogens and intestinal microbes, maintaining the dynamic balance of intestinal mucosa, and regulating the microbial-host immune response. In patients who have undergone an ileostomy, the number of goblet cells in the distal intestinal mucosa is significantly reduced relative to the proximal intestinal mucosa (25), and our studies also showed that the mucus layer of the distal intestinal mucosa is significantly thinner than that of the proximal intestinal mucosa (Figure 2). In mice with PN, although no changes were observed in total goblet cell numbers when compared with normal mice, levels of MUC2, RELMβ, and TFF3 in the ileum and luminal fluid were reduced (26). Similar to goblet cells, Paneth cells are specialized secretory epithelial cells resident in the small intestinal crypts of Lieberkühn (27). These cells secrete many dense granules such as lysozyme, secretory phospholipase A2 (sPLA2), α-defensins (cryptdins in mice), TNF, IgA, and RegIII, all of which balance the microbial population and mediate the inflammatory response to maintain intestinal homeostasis (27–29). These cells also secrete factors that help maintain and modulate the epithelial stem cells and progenitor cells that cohabitate in the crypts, which suggests that Paneth cells may play a major role in epithelial damage and repair (27). After an ileostomy, the Paneth cells in the distal intestinal mucosa of humans and mice are significantly decreased (3, 4) (Figure 2). In addition, following PN, there is reduced Paneth cell gene expression, such as sPLA2, and luminal levels of the proteins are also decreased (30). These results indicate that luminal stimulation may affect the proliferation, differentiation, and function of the goblet cells and Paneth cells, thereby affecting the chemical barrier.

The intestinal mucosal mechanical barrier generally consists of intestinal epithelial cells and tight junction proteins that connect them. As mentioned above, the distal dysfunctional intestine is atrophic; therefore, damage to the mechanical barrier is predictable. In addition, in a mouse model of colostomy, levels of occludin and E-cadherin were reduced in the distal dysfunctional intestinal segment, and the expression of claudin-3 was also decreased (31, 32). Regarding permeability, the absorption of fluorescein sodium and the permeability for mannitol and human serum albumin increased in the distal dysfunctional intestinal segment (33). These results indicate that the loss of luminal stimulation may lead to the reduction of tight junctions, which increases the permeability of the intestinal mucosa. Such destruction of the mechanical barrier is similar to that caused by PN; there are many reports on the destruction of the mechanical barrier caused by PN (34–36). As a result, some harmful substances, such as lipopolysaccharides (LPS), can easily enter the circulation through the intestinal barrier and cause abnormal reactions elsewhere in the body.

The immune barrier mainly consists of gut-associated lymphoid tissue (GALT), which consists of lymphocytes and lymphoid follicles in the intercellular space of the intestinal epithelium, lamina propria, and Peyer’s patches (37). Under normal conditions, approximately 70% of the total active immune cells in the body participate in this compartment (38). GALT plays a significant role in the elimination of foreign pathogens through the intestinal mucosa, maintenance of intestinal immune tolerance, regulation of gut microbiota, and maintenance of intestinal homeostasis (37, 39, 40). In a study on patients who have undergone an ileostomy, compared with the proximal functional intestinal mucosa, a significant decrease in the number of CD3 lymphocytes spontaneously secreting IFN-γ was observed in the distal dysfunctional mucosa among both intestinal intraepithelial lymphocytes (*p* = 0.008) and Lamina propria lymphocytes (*p* = 0.007). Although less significant, similar results were obtained for IL-4, especially in lamina propria lymphocytes (41). Another study also showed ileostomy-induced immune modulation with *a* > 50% decrease in activated T cell numbers and an increase in that of regulatory T cells in patients with steroid-resistant acute graft-versus-host disease of the gastrointestinal tract (42). Consistent with this, a lack of luminal stimulation reduces the contact between the immune barrier and foreign antigens, which weakens the ability of the adaptive immunity. Similarly, PN results in a series of dramatic changes in the GALT compartment, such as decreased levels of Th2 cytokines, production and release of IgA on mucosal surfaces, and cellularity and function of lymphocytes in the Peyer’s patch and lamina propria (43–45). Meanwhile, innate immune cells, such as neutrophils, accumulate and become primed, which may promote an inflammatory response (46). With these changes (Figure 2), the capacity for tolerance and adaptive response to previously encountered antigens of the intestinal barrier are reduced, leading to aggravated proinflammatory responses after injury, which seem to be associated with diversion colitis. Diversion colitis is a common non-specific inflammation of the malfunctioning bowel segment after diversion of feces, and intestinal immune disorders are an important risk factor (47). Loss of luminal stimulation seems to alter the immune balance of the distal dysfunctional mucosa and even of the rest of the body; this can temporarily relieve some intestinal immune diseases but may also cause intestinal inflammation in the long term. The biological barrier is not only affected by luminal stimulation, but is also part of the luminal stimulation which is discussed in the following sections in detail.

## Changes in Intestinal Microbes

Gut microbes consist of approximately 10^13^ bacterial cells of more than 250 different species, as well as various fungi, viruses, and archaea, and comprise the largest symbiotic microbial community in humans (48). Due to long-term coevolution, such an abundant gut microbiota has become an integral part of the host and plays a significant role in nutrition absorption and metabolism, biological antagonism, activation and promotion of intestinal immunity, and maintenance of intestinal homeostasis, which have been described in detail previously (49–51). Due to the different local intestinal environments, the microbes in different parts of the intestines show different compositions and diversity, and most of the intestinal microbes are concentrated in the distal intestines. Toward the distal end of the digestive tract, the oxygen concentration in the intestinal cavity shows a downward trend, and the distal ileum and lower intestinal segments are basically in an anaerobic environment (52). Therefore, gut microbes are mainly anaerobic bacteria such as Firmicutes (60–80%), Bacteroidetes (20–40%), Proteobacteria, and Actinobacteria (53, 54). However, an ileostomy destroys the intestinal continuity and exposes the intestinal cavity to the external environment, which changes the intestinal microenvironment and affects the gut microbes.

The survival of intestinal microbes depends on intestinal food contents and some endogenous secretions from the host, such as goblet cell mucin glycoproteins (55). Nevertheless, ileostomy dramatically changes the luminal environment of the distal ileum, which inevitably leads to microbial dysbiosis. *Via* a 16S rRNA gene analysis of the intestinal contents at both ends of ileostomy, Beamish et al. found that both the total mucosal bacterial load and microbial diversity were significantly reduced in the distal dysfunctional ileum compared with the proximal functional ileum (56). There was a significant reduction in the relative abundance of the Firmicutes phylum and a significant increase in that of the phyla γ-Proteobacteria and Bacteroidetes in the dysfunctional ileum when compared with the paired functional controls (56). The same changes were observed in the intestines of patients with PN (57), which is associated with intestinal inflammation (56). Furthermore, microbiome-metabolomic analysis of ileostomy indicated that the intestinal flora changed from strict anaerobes to facultative anaerobes before and after ileostomy, and returned to strict anaerobes after the reversal of ileostomy, suggesting that ileostomy permits oxygen entry in the lumen and promotes changes in intestinal microbial composition (58).

The reduction of bacterial load and microbial diversity may result in decreased competitive exclusion of pathogens, and an aerobic environment can stimulate the growth of certain pathogenic microbes. Previous studies have indicated that intestinal microbes are a factor leading to the occurrence and result of anastomotic leakages, and oral non-absorbable antibiotics can reduce but do not eliminate anastomotic leakage rates (59, 60). During the healing process of anastomotic tissues, some intestinal microbes, such as *Bacillus subtilis* strain (aerobic bacteria), *Enterococcus faecalis* (facultative anaerobes), and other facultative anaerobes produce a collagenase that can break down intestinal tissues at a rate seven orders higher than intestinal tissue collagenase. This is therefore likely to alter the dynamics of healing. These microbial-derived collagenases further trigger the production of matrix metalloproteinase 9 in intestinal tissues, which by itself possesses tissue-destruction capacities (61, 62). As a result, the process of healing that carries pathogenic bacteria becomes complex and can readily fail. In addition, microbial dysbiosis increases the risk of pathogen infection. After an ileostomy, a rare intestinal infection caused by methicillin-resistant staphylococcal enteritis has appeared clinically. Following PN, some opportunistic pathogens commonly related to infection, such as *E. coli*, *Salmonella*, *Vibrio*, and *Yersinia*, were observed (63). Compared with enteral feeding, experimental exposure of intestinal *C. albicans* during PN leads to increased colonization, mucosal translocation, and disseminated systemic infection (64). There is also a decrease in T cell numbers and IgA levels, as mentioned above. Fundamentally, the decline of intestinal mucosal immunity and the reduction of bacterial load and microbial diversity complement each other. As a result, pathogenic bacteria have a chance to grow, and abnormal immunity is activated.

In addition, the large intestine contains the largest bacterial ecosystem in the human body, constituting more than 70% of the symbiotic microbes in the body. The intestinal flora is usually discussed in the context of the disease state, which generally refers to the colonic flora (especially the flora derived from fecal metagenomic data) (65). The number of bacteria in the upper digestive tract is approximately 10^1^–10^2^ CFU/mL; in the ileum, about 10^4^–10^8^ CFU/mL; and in the distal colon, approximately 10^10^–10^12^ CFU/mL (66). Many previous studies have shown that intestinal microbes and their metabolites are directly or indirectly related to the pathophysiology of various body systems (Table 1). From this perspective, ileostomy results in the loss of nearly 70% of symbiotic microbes, which means that ileostomy significantly affects all systems of the body. Intestinal microbes are closely related to various conditions, such as cardiovascular disease, obesity, and motor system diseases. Nevertheless, there are few reports on the abnormal outcomes of various systems after ileostomy. Clinical research on ileostomy has shown that patients have reduced bone mineral density and are more likely to have brittle fractures after surgery (67, 68). It has been confirmed that gut microbes play a central role in maintaining bone health and influencing bone turnover and density. Gut microbes indirectly regulate the absorption of calcium and the production of intestinal serotonin by regulating the immune system with SCFAs. Serotonin is a molecule that interacts with osteoblasts and is considered to be a bone quality regulator that regulates bone metabolism (69). Gut microbes are also involved in the bile acid metabolism, and the bile acid index of patients who have undergone an ileostomy appears to be abnormal based on our clinical observation. These results, however, indicate that gut microbes do not seem to be essential for survival. The body’s strong compensatory ability may make up for the lack of intestinal microbes in a relatively short period. Further elucidation of the effects of the gut microbes on the body should provide more helpful insight on the postoperative recovery and prevention of further complications in patients.

**TABLE 1:** The microbial metabolites and compositions associated with human systems.

System	Microbial related substances	Disease (references)
Circulatory system	Lipopolysaccharides (LPS), trimethylamine (TMA), bile acids, short-chain fatty acids (SCFAs), phenylacetic acid, p-cresyl sulfate, indoxyl sulfate, anthocyanins, phytoestrogens	Cardiovascular disease (82)
Motor system	SCFAs, LPS medium chain fatty acids (MCFAs), tryptophan and tryptophan-derived metabolites, polyamines, TMA	Spondyloarthritis (83), Osteoporosis (84), Osteoarthritis (85)
Endocrine system	SCFAs, γ-aminobutyric acid (GABA), circulating branched-chain amino acids (BCAAs), serotonin (5-HT), other neurotransmitters (NTs), LPS, trimethylamine N-oxide (TMAO), tryptophan-derived indoles, bile acids	Obesity (86), Type 2 diabetes mellitus (87)
Nervous system	SCFAs, GABA, 5-HT, other NTs, amyloids, LPS, histamine, dopamine, endotoxin, hydrogen, hydrogen sulfide (H_2_S), indoleacetic acid	Alzheimer’s Disease (88), Parkinson’s disease (89)
Digestive system	LPS, SCFAs, urolithins, bile acids, tryptophan, succinate, ethanol, H_2_S, polyamines, toxins	Cancer (90), Inflammatory bowel disease (91)
Urinary system	Tryptophan, SCFAs, LPS, TMAO, Phenols, indole	Kidney disease (92, 93)
Respiratory system	LPS, SCFAs, microbe-associated molecular patterns	Chronic Obstructive, Pulmonary Disease, Asthma, Cystic Fibrosis (94, 95)

## Changes in Smooth Muscle and Bowel Motility

The intestinal smooth muscles mainly consist of longitudinal and circular smooth muscles, which generate coordinated motility patterns to promote full digestion, absorption, and emptying of intestinal contents. Such coordinated motility of the intestine is not only under multiple levels of control, including the enteric nervous system (ENS), central nervous system (CNS), intestinal hormones, and paracrine agents, but is also related to other factors such as gut microbes and mechanical stimulation of intestinal contents (70, 71). Consideration of ileostomy may provide some insight.

After an ileostomy, the intestinal motility of the distal dysfunctional intestinal segment is markedly decreased, which may be an important cause of postoperative ileus following the reversal of ileostomy (72, 73). The postoperative ileus is the most common complication after the reversal of ileostomy; the reported incidence is usually between 15 and 32% (74). What leads to a loss of intestinal motility? From the perspective of effectors, similar to the atrophy of the intestinal mucosa, the strength and area of intestinal smooth muscle are both significantly reduced (6) (Figure 2). Disuse atrophy may result from the loss of mechanical stimulation of the intestinal contents. From the perspective of the controller, in the bypass ileum of the rat model, the number of neurons expressing pituitary adenylate cyclase-activating peptide, neuropeptide Y, or vasoactive intestinal peptide decreased gradually, and the number of neurons expressing nitric oxide synthase increased significantly, especially in the intermuscular ganglion (75). In rabbits with a colostomy, the nitrergic-peptidergic, cholinergic-peptidergic, and non-cholinergic–non-nitrergic responses in the distal colonic segment were significantly decreased compared with those in the control group (76). Neural activities related to contraction and relaxation seem to be reduced, but the relaxation responses are relatively stronger, which may contribute to the hypomotility noted in inactive intestines. From the perspective of regulators, gut microbes influence intestinal motility in a variety of ways; for instance, gut microbes indirectly affect intestinal motility by producing metabolites, such as SCFAs, and peptides to stimulate the ENS, or by influencing the production of serotonin (Figure 2), and they can also directly affect intestinal motility through Toll-like receptors (the bacterial recognition receptors) expressed by smooth muscle cells (70, 71, 77). The relationship between intestinal motility and gut microbes has previously been elaborated, but there are still many questions left to answer (78). In addition, bile acid and mucus also participate in the regulation of intestinal motility, which suggests that the loss of bile acid and mucus may be an important cause of the hypomotility noted in the distal dysfunctional intestinal segment (78). Disturbance of intestinal homeostasis also leads to abnormal motility of the intestines.

Based on the factors mentioned above, a new method for stimulation of the distal dysfunctional intestinal segment with probiotics and prebiotics before the reversal of ileostomy has been attempted in clinical practice (79–81). The results showed that it promotes the recovery of postoperative intestinal function and reduces the incidence of ileus; however, there is insufficient high-quality evidence to recommend routine implementation of preoperative bowel stimulation in clinical practice. Questions on the target and period of stimulation still require further research.

## Concluding Remarks and Future Perspectives

An ileostomy is an operation with both benefits and risks, and effective promotion of postoperative recovery and prevention of complications have been unsolved clinical problems for a long time. Understanding the physiological and pathological changes in patients who have undergone an ileostomy will help solve these. Previous studies have shown that intestinal stimulations, such as mechanical stimulation and that from intestinal microbes, have various effects on the intestinal mucosa. Based on this, some medical measures, such as postoperative enema with probiotics or normal saline, have been tried in the clinic, but their effects have not been satisfactory. The postoperative state of patients who have undergone an ileostomy is complex and requires a standardized evaluation system. More evidence-based medicine studies are needed to establish effective and standardized postoperative interventions for ileostomy.

In addition to focusing on the clinical issues that are related to an ileostomy, the conditions of an ileostomy can be ideal to study various experimental questions. As the colon is left unused for a long time, the metabolism of some drugs or foods in the small intestine can be studied by testing the stool of the small intestine. Generally, the ileostomy is reversed after half a year, and the distal intestinal microbes undergo a process from loss to reconstruction during this period. Microbiomics can be used to understand how the microbes at the distal intestinal segment are naturally rebuilt; this will further contribute to the study of the dynamic evolution of gut microbes. Additionally, based on the influence of gut microbes on body metabolism, studying this process using metabolomics linked to microbiomics can further elucidate the relationship between metabolism and gut microbes. This will, in turn, provide more valuable suggestions for postoperative recovery and prevention of complications after ileostomy. Of course, such questions can also extend to colostomy and the relationship between ileostomy and cancer and inflammatory bowel disease. Answers to some of these questions may not only explain some normal physiological changes but also help solve clinical problems related to fistulas.

## Author Contributions

HM conceived and wrote the manuscript. YQ and WX contributed to the writing and revision of the manuscript. XL and HY participated in the discussion of issues related to ileostomy. All authors contributed to the article and approved the submitted version.

## Conflict of Interest

The authors declare that the research was conducted in the absence of any commercial or financial relationships that could be construed as a potential conflict of interest.

## Publisher’s Note

All claims expressed in this article are solely those of the authors and do not necessarily represent those of their affiliated organizations, or those of the publisher, the editors and the reviewers. Any product that may be evaluated in this article, or claim that may be made by its manufacturer, is not guaranteed or endorsed by the publisher.
